# Measurement of Coagulation Factor Activity and Bacterial Growth in Canine and Feline Fresh Frozen Plasma With Unintended Exposure to Room Temperature

**DOI:** 10.1111/vec.70154

**Published:** 2026-08-31

**Authors:** Samantha J. Bilko, Julie M. Walker

**Affiliations:** ^1^ University of Wisconsin–Madison Madison Wisconsin USA

**Keywords:** labile, transfusion, von Willebrand factor

## Abstract

**Background:**

To assess the viability of canine and feline plasma for the purpose of transfusion therapy after freezer malfunction and inadvertent thawing of numerous fresh frozen plasma (FFP) units resulted in prolonged exposure to room temperature.

**Key Findings:**

After a plasma freezer alarm malfunction, numerous units of canine and feline FFP thawed over a period of approximately 10 h. Hemostatic capacity was assessed with measurement of prothrombin time (PT), partial thromboplastin time (PTT), and coagulation factor analysis on a representative unit of canine and of feline FFP. Analysis of samples demonstrated normal PT and PTT, with all coagulation factor activities exceeding 70% of reference interval standards. No bacterial growth was noted after this duration of time.

**Significance:**

Hemostatic capacity of representative canine and feline FFP units appeared to remain normal and may be considered a viable transfusion product after exposure to room temperature for up to 10 h and within the circumstances described in this report.

**Abbreviations**
FFP: fresh frozen plasma
PT: prothrombin time
PTT: partial thromboplastin time
pRBC: packed red blood cell
vWF: von Willebrand factor

## 1 Introduction

Fresh frozen plasma (FFP), a valuable blood bank resource, is essential in the treatment of critically ill patients with conditions including coagulopathy, hemorrhage, hypoproteinemia, and endothelial glycocalyx dysfunction [1, 2]. To meet standards set by the Association for the Advancement of Blood and Biotherapies, FFP is separated from whole blood within a defined time period from collection and can be stored in a freezer −18°C or colder [3]. When stored according to these guidelines, FFP maintains therapeutic concentrations of all functional hemostatic proteins, albumin, and globulins [4, 5].

To prepare for transfusion, plasma should be thawed in an appropriate plasma thawing device or water bath (temperature between 30°C and 37°C), then transfused immediately or kept in a refrigerator at 1°C–6°C for no more than 5 days before use [3]. Alternatively, one veterinary study demonstrated that both canine and feline FFP units maintain hemostatic capacity after sustaining at least one freeze–thaw cycle [6]. More recent literature suggests that thawed canine FFP maintains therapeutic concentrations of clotting factors, including heat‐labile factors V and VIII, through prolonged storage under refrigeration or at room temperatures [7, 8]. Similar findings have not been published for feline product. The purpose of this report is to describe the hemostatic capacity and assess for bacterial growth in representative units of canine and feline FFP after an inadvertent freezer malfunction led to the contents of a blood bank freezer being exposed to room temperature for up to 10 h.

## 2 Materials and Methods

Approximately 11 units of canine FFP, seven units of feline FFP, and five units of feline frozen plasma were stored for clinical use in a commercial blood bank freezer^1^ at a university teaching hospital. All units were collected and processed using aseptic technique according to institutional protocols [9]. The plasma units were stored at −30°C until the freezer door was inadvertently left open overnight and the units were found in a completely liquid state the following morning, up to 10 h later. Upon individual examination and handling, each unit had subjectively equilibrated with the temperature of the room (20°C). One canine FFP unit (sourced from the in‐house blood donor program) and one feline FFP unit (sourced from an outside veterinary blood bank^2^) located centrally within their respective drawers were randomly selected from the thawed plasma pool for testing. Using examination gloves and aseptic technique, a sterile intravenous bag spike with a needleless connector was inserted into one of the bag's spike ports. The Luer adapter surface was cleaned with isopropyl alcohol and allowed to dry. A sterile syringe was used to withdraw a sample of plasma from the unit for coagulation testing and culture. Prothrombin time (PT) and partial thromboplastin time (PTT) were measured using an in‐house analyzer^3^. Bacterial culture was performed under a laminar flow hood on each unit by inoculating plasma into aerobic and anaerobic culture media^4^, which were then incubated under controlled conditions^5^ for 5 days. A new, sterile needle was used to inoculate each vial with 5 mL of plasma. An additional 10 mL of plasma was collected from each unit and refrozen in 0.5‐mL aliquots at −30°C. These were submitted for factor XIII deficiency screening and measurement of fibrinogen, coagulant activity of factors II, V, VII, VIII, IX, X, XI, and XII, and von Willebrand factor (vWF) at an outside laboratory^6^.

## 3 Results

Both canine and feline plasma samples had normal PT and PTT times (Table 1). Factor XIII deficiency screening was normal for both plasma units, indicating at least 5% factor XIII activity. All coagulant factor activities remained within or exceeded the reference intervals (Table 2). In the feline sample, the factors with the lowest activities were VIII (76%) and vWF (77% as assessed via collagen binding assay). Results of all factor analyses exceeded 100% in the canine plasma sample. Bacterial cultures for both units were negative after incubation for 5 days.

**TABLE 1:** Results of prothrombin time (PT) and partial thromboplastin time (PTT) from representative samples of canine and feline plasma after exposure to room temperature for approximately 10 h. All values in seconds.

	Canine plasma sample	Canine reference interval	Feline plasma sample	Feline reference interval
PT	7.9	7–9.4	11.9	8.7–12.6
PTT	12.3	8.5–13.8	16.2	8.9–17.4

**TABLE 2:** Coagulation factor analysis results in representative samples of canine and feline plasma after exposure to room temperature for approximately 10 h.

Analyte	Canine factor activity	Feline factor activity	Reference interval (canine and feline)
Factor II	137%	127%	50%–150%
Factor V	122%	157%	50%–150%
Factor VII	161%	102%	50%–150%
Factor VIII	175%	76%	50%–200%
Factor IX	177%	102%	50%–150%
Factor X	123%	104%	80%–175%
Factor XI	211%	165%	60%–150%
Factor XII	205%	119%	60%–150%
Fibrinogen	304 mg/dL	401 mg/dL	76–270 mg/dL
vWF (vWF:Ag)	149%	97%	70%–180%
vWF (vWF:CBA)	134%	77%	50%–170%

Abbreviations: vWF, von Willebrand factor; vWF:Ag, vWF antigen measured via enzyme‐linked immunosorbent assay; vWF:CBA, vWF collagen binding assay.

## 4 Discussion

This report describes the investigation that was performed after canine and feline units stored in a university blood bank freezer were accidentally exposed to room temperature conditions for up to 10 h. Representative plasma samples were tested for coagulant factor activity and to verify that bacterial growth had not occurred under these conditions. The authors performed these tests to assess the safety and utility of the remaining plasma units for species‐specific administration to clinical patients, rather than downgrading or discarding the remaining product.

The findings presented in the current report are consistent with the results of a recent ex vivo study that demonstrated stability of factors V, VII, VIII, and IX, and lack of bacterial growth when 20 units of canine FFP were held at room temperature for 12 h during simulated constant rate infusion [7]. Similarly, both canine and feline units tested here demonstrated acceptable coagulation factor activities and vWF concentration. Neither product demonstrated bacterial growth.

A general quality assurance guideline for plasma products in human medicine is that 1 mL of FFP contains approximately 1 unit of coagulation factor activity. This has been adopted in veterinary medicine, where individual factor activity is reported as a percentage value, representing the units of activity per milliliter (U/mL). This is compared with a pooled canine plasma standard, which has an assigned value of 1 U/mL, or 100% [5]. A European standard for FFP quality control is based on labile factor VIII activity of no less than 0.7 U/mL, or 70% [10]. Similarly, in veterinary medicine, factor VIII undergoes significant degradation in canine and feline FFP when faced with temperature variability or prolonged storage [4, 5]. Other than factor VIII, the factors demonstrating the lowest activities in feline FFP after 1 year of storage were VII and XII [4], while the factors with the lowest activities in stored canine FFP after 1 year were IX and X [5]. In a study of canine FFP held at room temperature for 12 h during simulated constant rate infusion, the authors did not demonstrate a reduction in factor V, VII, VIII, or IX activity after 4 or 12 h of exposure to room temperature [7]. In the data presented in this study, factor VIII and all other factor activities were within the acceptable range, with only feline factor VIII demonstrating activity below 100%. PT and PTT testing remained within normal range for each species, suggesting therapeutic adequacy for treating coagulopathy.

The current report adds to the growing body of evidence surrounding the validity of the “4‐h rule” for transfusing blood products. This recommendation in human medicine originates from transfusion‐associated sepsis complications, more typically associated with platelet products due to their preparation with additive solutions containing more energy substrates and storage at higher temperatures than packed red blood cells (pRBCs) or plasma [3, 11]. However, this “rule” has been extrapolated to include pRBCs, FFP, and other component therapy in both human and veterinary medicine. In a systematic review of exposure times in pRBC units, the authors reported bacterial growth in five of 648 (0.8%) units that sustained room temperature exposure for approximately 6 h, with four of six evaluated studies finding no bacterial growth in any tested units [12]. Among the studies reviewed, the length of time and degree of temperature exposure varied from hours to up to 1 day, making it difficult to draw meaningful conclusions about a specific safe or unsafe time frame. Additionally, the quality of evidence and the age of the studies were limiting, as blood collection and storage techniques have evolved over time. These limitations left the authors unable to make a strong clinical recommendation, although they offered a suggestion that the “4‐h rule” for pRBCs could be extended to 5 h or longer without significant detriment to blood quality [12]. However, given the serious clinical implications of transfusing pRBCs that could be contaminated, this comment should be interpreted cautiously until more robust conclusions can be documented. Few data exist within the veterinary literature, but a recent multi‐institutional surveillance study reports that 19% of canine pRBC transfusions administered exceeded the 4‐h time limit with no evidence of transfusion‐associated sepsis in any patient [13]. Mesa‐Sanchez et al. evaluated canine FFP for evidence of bacterial growth after 4 and 12 h of exposure to room temperature and found no evidence of contamination [7]. Our report is the first to demonstrate the sterility of a feline FFP unit after being exposed to room temperature for a similar period of time.

Limitations to this investigation include the absence of baseline coagulation factor activity in the analyzed units, making it impossible to quantify the decline in clotting factor activity, as well as the precise timing of exposure of the affected units being unknown. The rate of thaw may have been faster or slower depending on the extent to which the freezer door was left ajar and the exact placement of units within the freezer, which in turn may have affected the degree of product degradation. Representative units were tested, rather than compromising the integrity of all units; however, it is possible that results may have differed among individual units, had they all been tested. These limitations make it difficult to extrapolate these data to clinical use, such as when administering FFP infusions over a time frame greater than 4 h.

In conclusion, coagulation factor activity and sterility were maintained in two canine and feline units after exposure to room temperature conditions for up to 10 h. Performing similar analyses on representative units might be useful to blood banks or hospitals, which may experience freezer malfunction, to indicate that product may not need to be downgraded or discarded. A larger controlled study is warranted to support these results.

## Author Contributions

**Samantha J. Bilko**: conceptualization, investigation, writing – review and editing, writing – original draft. **Julie M. Walker**: conceptualization, investigation, writing – review and editing, supervision.

## Ethics Statement

The authors confirm that the ethical policies of the journal, as noted on the journal's author guidelines page, have been adhered to. No ethical approval was required as no live animals were used in this study.

## Conflicts of Interest

The authors declare no conflicts of interest.

## Endnotes

1 −30°C Plasma Storage Freezer (iPF125), Helmer Scientific, Noblesville, IN.

2 Animal Blood Resources Inc., Stockbridge, MI.

3 STA Compact Max, Stago Diagnostica, Asnières‐sur‐Siene, France

4 BD BACTEC Plus Aerobic/F and BD BACTEC Lytic/10 Anaerobic/F Culture Vials, Becton, Dickinson and Company, Sparks, MD.

5 BD BACTEC FX40 Blood Culture System, Becton, Dickinson and Company, Franklin Lakes, NJ.

6 Comparative Coagulation Laboratory Animal Health Diagnostic Center, Cornell University, Ithaca, NY.
